# A Novel Tannic Acid-Based Carbon-Supported Cobalt Catalyst for Transfer Hydrogenation of Biomass Derived Ethyl Levulinate

**DOI:** 10.3389/fchem.2022.964128

**Published:** 2022-07-11

**Authors:** Meng Wang, Xuefeng Yao, Yuxin Chen, Baodong Lin, Na Li, Keduan Zhi, Quansheng Liu, Huacong Zhou

**Affiliations:** College of Chemical Engineering, Inner Mongolia University of Technology, Hohhot, China

**Keywords:** biomass, tannic acid, carbon support, ethyl levulinate, hydrogen transfer, catalyst

## Abstract

The catalytic conversion of ethyl levulinate (EL) to γ-valerolactone (GVL) is an important intermediate reaction in the conversion and utilization of biomass resources. The development of novel and efficient catalysts is significantly important for this reaction. In this work, using the biomass-derived tannic acid as carbon precursor and the transition metal cobalt as active component, a novel tannic acid carbon supported cobalt catalyst (Co/TAC) was prepared by pyrolysis and subsequent hydrazine hydrate reduction method. The hydrogenation of EL and other carbonyl compounds by hydrogen transfer reaction was used to evaluate the performance of the catalysts. The effects of different preparation and reaction conditions on the performance of the catalysts were investigated, and the structures of the prepared catalysts were characterized in detail. The results showed that the carbonization temperature of the support had a significant effect on the activity of the catalyst for the reaction. Under the optimized conditions, the Co/TAC-900 catalyst obtained the highest GVL yield of 91.3% under relatively mild reaction conditions. Furthermore, the prepared catalyst also showed high efficiency for the hydrogenation of various ketone compounds with different structures. This work provides a new reference for the construction of the catalysts during the conversion of biomass and a potential pathway for the high-value utilization of tannin resource.

## Introduction

With the rapid development of human society, the demand for carbon resources and the consumption of fossil resources are increasing. It is the goal of human society to find renewable carbon resources and improve the utilization efficiency of carbon resources. Biomass, as an abundant, inexpensive and renewable organic carbon resource, is considered as an ideal substitute for traditional fossil resources (Li et al., 2017a; Xu et al., 2020a). A promising way to realize the utilization of biomass is the efficient catalytic conversion of biomass into fuels and platform chemicals. Ethyl levulinate (EL) is one of the most important biomass platform molecules that can be further converted into various high-value products (Tuck et al., 2012). γ-Valerolactone (GVL) is a useful chemical derived from lignocellulose and has the advantages of high stability, low toxicity and safe storage. Additionally, it can be used to produce liquid fuels, polymers, intermediates in fine chemical production, and food additives, *etc*., (Wang et al., 2013; Han et al., 2014; Liguori et al., 2015). Due to its potential applications, GVL is considered as a bridge linking biomass and valuable chemicals.

Hydrogenation of EL to GVL is one of the key reactions for the catalytic conversion of sustainable biomass into value-added chemicals. In the synthesis of GVL from biomass, the noble metal based catalysts are considered as excellent catalysts for the hydrogenation of EL to GVL with molecular hydrogen as the hydrogen source, and a variety of noble metal-based homogeneous catalysts such as Ru, Pd, Pt, Ir, Re, and Rh, *etc*., have been reported for the conversion of EL to GVL. However, high-pressure hydrogen has considerable security risk. Additionally, the cost of noble metals is high and the resources are scarce, and ligand synthesis of homogeneous catalysts is complex and has poor recovery performance (Leitner, 2002; Manzer, 2004; Du et al., 2011; Yan et al., 2013a; Liu et al., 2013; Xin et al., 2014). In contrast, the synthesis of GVL from EL *via* the catalytic transfer hydrogenation (CTH), also known as Meerwein-Ponndorf-Verley (MPV) reaction, can realize the hydrogenation using liquid hydrogen such as isopropanol as the hydrogen source and non-noble metal based catalysts. Therefore, a series of non-noble metal based heterogeneous catalysts have been developed due to their superior performances (Gilkey and Xu, 2016; Osatiashtiani et al., 2017; Xu et al., 2020a), among which cobalt is an abundant metal with high availability and low price, and cobalt-based catalysts are widely used in the well-known Fischer-Tropsch synthesis and other hydrogenation reactions (Long et al., 2015; Osatiashtiani et al., 2017). Besides the metal active sites, the catalyst carrier has the function of supporting and dispersing metal active components. The supports reported in the literature for biomass transfer hydrogenation include Al_2_O_3_ (Long et al., 2015), zeolite (Obregón et al., 2014), SiO_2_ (Ye et al., 2014) and carbon materials, *etc.* Carbon materials have attracted much attention due to their wide availability, high mechanical strength, and good chemical stability (Yan et al., 2013b). It is worth noting that biomass-based carbon has a wide range of sources and good physical and chemical properties, making it an environmentally friendly carbon material and an excellent choice as the candidate for catalyst support (Luo et al., 2015).

Tannic acid (TA) is a renewable and environmental friendly natural functional molecule with rich carbon content and functional groups such as gallic alcohol or catechol groups, which can be extracted from plant tissue. These advantages of TA contributed to its wide applications (MacDermid-Watts et al., 2021). For example, metal-tannin coordination polymers have been widely used as multifunctional platforms for functional surface engineering (Huang et al., 2018). There were also various researches on the use of tannic acid in adsorbent, cross-linking agents, coating materials, and modifying polymer surfaces (Rubentheren et al., 2015; Zhang et al., 2017). TA was also used in the preparation of catalysts. Since the phenolic hydroxyl groups in tannic acid have a strong chelating effect with metal ions, they can be used as ligands to coordinate with metal ions to prepare coordination catalysts. Many previous studies have demonstrated that tannic acid can coordinated with metal ions such as Zr, Hf, Ni, Fe, Cr, Cu, Rh, *etc*., for furfural hydrogenation and photoelectric catalysis (Mao et al., 2012; Choudhary et al., 2017; Shi et al., 2018; Xu et al., 2020a). TA also has great potential in the preparation of metal/carbon composites due to its strong chelating ability with metal ions (Wang et al., 2020). Carbon materials are generally prepared from tannic acid by pyrolysis. There were some reports on the application of carbon supports produced by the pyrolysis of tannins for ORR, OER and HER electrocatalysts (Cao et al., 2020). The tannin-based carbon supported metal catalysts were used into the catalytic conversion of biomass. Varila et al. (Qin et al., 2019) reported the conversion of furfural to furfuryl alcohol with a Cu/Ni bimetallic catalyst supported by condensed tannin-based carbon foam, and achieved a high conversion of furfural at 503 K and 40 bar H_2_. Cobalt-based catalysts are excellent catalysts for biomass transfer hydrogenation using isopropanol as hydrogen source and solvent. Most of the reported supported catalysts prepared from cobalt and tannic acid were used for the hydrogenation of the halogenated aromatic or the degradation of dyes (Wang et al., 2020; Choi et al., 2021; Varila et al. (2021). To the best of our knowledge, the direct use of tannic acid derived carbon supported transition metal cobalt catalysts for the CTH reaction of EL has not been reported.

**SCHEME 1:**
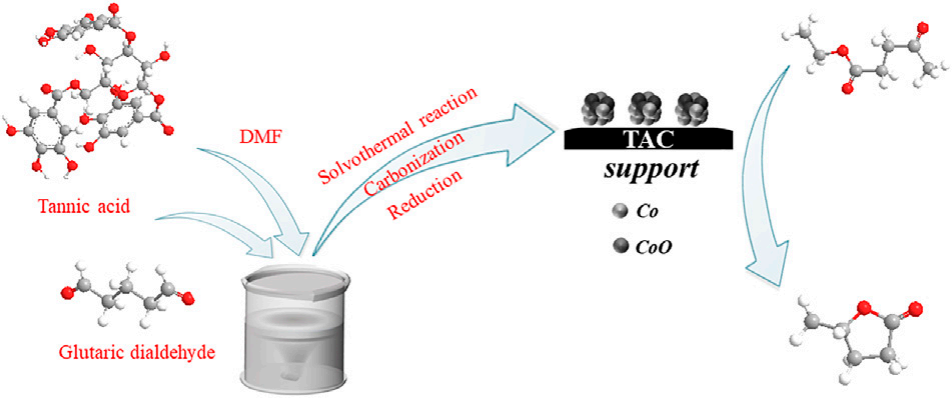
Synthesis routes of the Co/TAC catalysts from tannic acid and reaction route for EL hydrogen transfer over Co/TAC catalysts.

Herein, we prepared high-performance tannic acid carbon-supported cobalt catalyst by direct pyrolysis of TA and then hydrazine hydrate reduction method. The performance evaluation of the catalysts was carried out using the CTH reaction of EL to GVL (Scheme 1). The results showed that the carbonization temperature of the support had a significant effect on the catalytic activity, 94.3% conversion of EL and a GVL yield of 91.3% were achieved at the reaction temperature of 150°C for 5 h. Co^0^ played a key role in the hydrogen transfer reaction and cooperated with the acid-base sites in the catalyst to promote the reaction. The prepared catalyst had a good catalytic performance for ketone compounds with different structures. This work is of great significance for the development of efficient catalysts in the catalytic conversion of biomass.

## Experimental

### Materials

Tannic acid (TA, 98%), glutaraldehyde solution (50%), ethyl levulinate (EL, 98%), isopropyl alcohol (iPrOH, 99.5%) were purchased from Beijing Innochem Technology Co. Ltd. Cobalt (Ⅱ) chloride (CoCl_2_ 6H_2_O), glycol, sodium hydroxide were A.R. grade and provided by Tianjin Fengchuan Chemical Reagent Technology Co. Ltd. γ-Valerolactone (GVL, 99.5%) and decane were obtained from Beijing J&K Scientific Ltd.

### Preparation of Carbon Support

The preparation of the carbon support using TA in this work was as follows. TA (1 mmol) was dissolved into DMF (20 ml) and stirred for 1.5 h at room temperature. Then, 25 ml 50% glutaraldehyde solution was added into tannic acid solution and stirred under room temperature for 30 min. The mixed solution was transferred to the PTFE lining of the hydrothermal reactor, and solvothermal reaction was carried out at 200°C for 3 h in a Muffle furnace. After solvothermal reaction, the viscous polymer was dried at 100°C and heated to the desired temperature (300–900°C) under nitrogen atmosphere at a heating rate of 10°C/min for 2 h. After being ground in agate mortar, the black powders were collected. The obtained TA-derived carbon support was denoted as TAC-T (T referred to the carbonization temperature).

### Preparation of Catalysts

The Co/TAC-T catalysts were prepared by hydrazine hydrate reduction method. In a typical process, 1.0 g TAC and 3.0 g NaOH were added into the mixture of 20 ml water and 20 ml glycol in breaker A. 1.0 g CoCl_2_.6H_2_O was added into 20 ml glycol in breaker B and stirred for complete dissolution. Beaker A was placed in a water bath with magnetic stirring at 25°C and then 40 ml 80% (v/v) hydrazine hydrate was added to beaker A. The CoCl_2_.6H_2_O in beaker B was slowly dropped into beaker A with a separatory funnel solution and vigorously stirred for 3 h. The obtained solid precipitate was washed with distilled water until the pH was neutral and dried in vacuum at 80°C for 12 h.

### Catalyst Characterization

Scanning electron microscope (SEM) measurements were performed on a Hitachi S-4800 scanning electron microscope operated at 15 kV. High resolution transmission electron microscope (HRTEM) images were obtained using a TEM JEOL-1011 with an accelerating voltage of 120 kV. The high-angle annular dark-filed scanning TEM (HAADF- STEM) images were performed on a Talos F200X instrument. The specific surface area, pore volume and average pore diameter were measured on a 3H-2000PS2 type analyzer (Beishide instrument Co. Ltd.) by nitrogen adsorption-desorption method. Samples were pretreated for 8 h under vacuum at 250°C before testing. X-ray diffraction (XRD) patterns were recorded on XD8 Advance-Bruker AXS X-ray diffractometer using Cu-Kα radiation (λ = 0.1543 nm) at 40 kV and 40 mA. The scanning speed was set to 30°/min ranging from 5° to 90°. X-ray photoelectron spectroscopy (XPS) test was performed on an ESCALAB 250Xi spectrometer (Thermo Fisher Scientific) equipped with Al kα excitation source (hν = 1486.6 eV) and operating at 15 kV and 150 W. Calibration of binding energy by reference to C1 s signal (284.6 eV). Raman spectra at 120 cm^−1^–4000 cm^−1^ (λ = 532 nm) were collected on a Renishaw inVia microscope. Temperature-programmed desorption of carbon dioxide (CO_2_-TPD) was performed on Micromeritics AutoChem II 2920. The samples were pretreated at 500°C for 1 h to remove physisorbed CO_2_ by flowing helium at 100°C. The strongly adsorbed CO_2_ was heated from room temperature to 800 °C at a rate of 10°C/min for desorption under the flow of helium. The temperature-programmed desorption of ammonia (NH_3_-TPD) was performed on Micromeritics AutoChem II 2920. The samples were pretreated at 500°C for 1 h, and the physically adsorbed NH_3_ was removed by He flowing at 100°C. Under helium flow, the strongly adsorbed NH_3_ was heated from room temperature to 800°C at a rate of 10°C/min for desorption. The content of Co element in the Co/TAC catalyst was determined by inductively coupled plasma-optical atomic emission spectroscopy (ICP-AES) on Thermo Fisher Scientific iCAP 7000.

### Catalytic Activity Test

The CTH reaction of EL and other ketones to produce the corresponding alcohol or its derivatives was carried out in a stainless steel closed reactor equipped with a polytetrafluoroethylene lining (15 ml in volume). In a typical reaction process, 1 mmol EL, 25–200 mg catalyst and 5 ml iPrOH were added to the lining. The reactor was sealed and purged with N_2_ several times to remove air. Then, the reactor was filled with 0.1 MPa N_2_ as a protective gas, and it was placed in an oil bath with magnetic stirring and reacted for 1–8 h at the temperature of 130–160°C. After the reaction, the reactor was rapidly cooled in an ice water bath, and the reaction solution and the catalyst were separated by centrifugation. The separated supernatant was analyzed by a gas chromatography (TECHCOMP GC7900) with flame ionization detector using decane as the internal standard. The identification of products and reactants was carried out on GC-MS (Agilent Technologies 7000D). The EL conversion, GVL yield and GVL selectivity were calculated using the following equations (Zhao et al., 2020):

$$EL\;conversion\left(\%\right)=\left(1-\frac{\text{Residual m}oleofEL}{InitialmoleofEL}\right)\times 100\text{\%}$$ (1)

$$GVL\;yield\left(\%\right)=\frac{\text{Generated }mole\;of\;GVL}{Initial\;mole\;of\;EL}\times 100\%$$ (2)

$$GVL\text{ selectivity}\left(\%\right)=\frac{GVL\;yield}{EL\;conversion}$$ (3)

$$TOF\left(h^{-1}\right)=\frac{Conversion\;mole\text{ of EL}}{\text{Mole of Co catalyst}\times \text{Reaction time}}$$ (4)

In the reusability experiment, the catalyst was separated from the reaction system by centrifugation and washed three times with isopropanol, then used for the next cycle without other treatments. All experiments during the experiment have been repeated at least twice. The quantitative analysis of reactants and products was repeated three times to ensure the accuracy of the data, and the average value was taken.

## Results and Discussion

### Activity of the Catalysts

**FIGURE 1:**
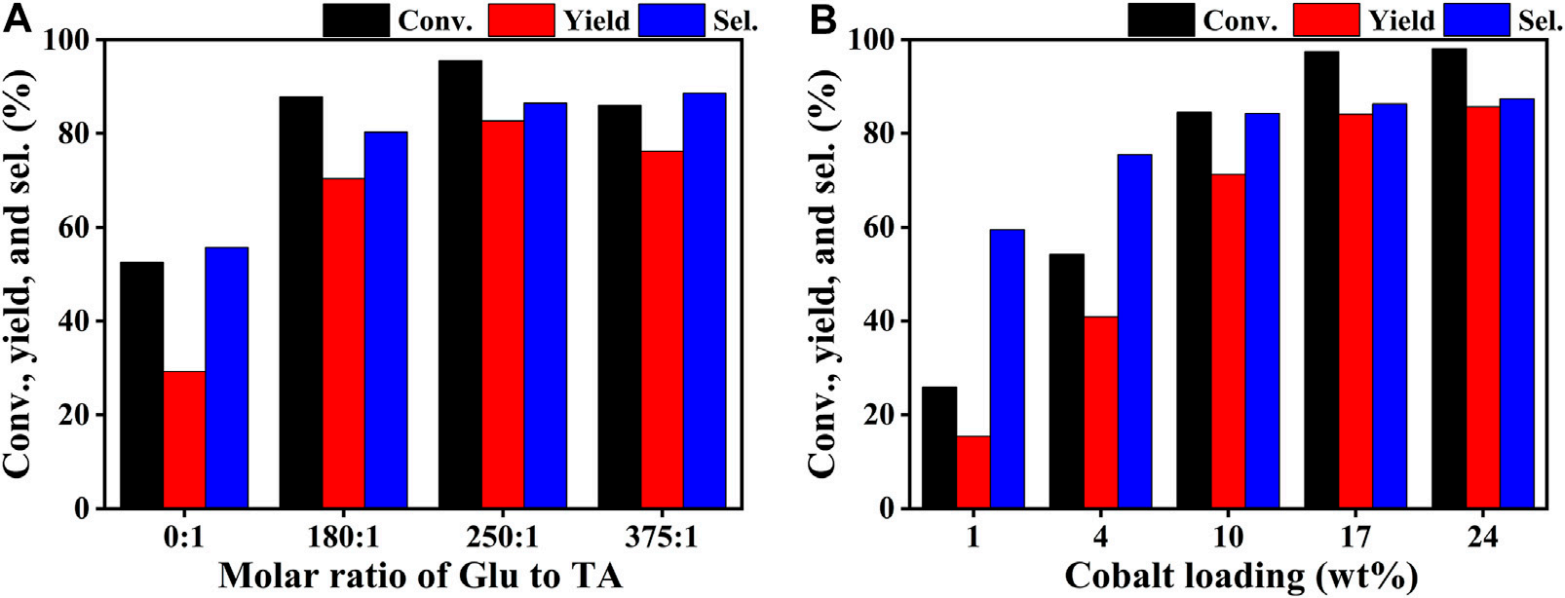
Influence of **(A)** molar ratio of glutaraldehyde (Glu) to TA and **(B)** Co loading on the catalytic performance of the catalyst. Reaction conditions: EL 1 mmol, Co/TAC 100 mg, iPrOH 5 ml, 0.1 MPa N_2_, reaction temperature 150°C, reaction time 3 h.

The catalytic performance evaluation of the Co/TAC-T catalysts for the CTH reaction of EL to GVL was carried out. As can be seen from Table 1, the reaction cannot proceed without any catalyst (entry 1). In the cases of TAC, CoCl_2_ 6H_2_O alone, and pure CoO as catalysts, it was observed that the conversion of EL was quite low or nearly zero (Table 1, entries 2–4). Comparatively, the prepared catalysts showed different activities for the reaction (Table 1, entries 5–9). The performance of the Co/TAC-T catalysts was optimized by changing the preparation conditions. Three variables, the ratio of glutaraldehyde to TA, the carbonization temperature and the cobalt loading were investigated. As the carbonization temperature increasing, the conversion of EL and the yield of GVL increased, and the highest conversion and yield were obtained on Co/TAC-900 (Table 1, entries 5–9). The effects of the ratio of glutaraldehyde to TA and the cobalt loading were shown in Figure 1. The catalytic performance of the Co/TAC catalyst prepared under the ratio of glutaraldehyde to TA 250:1 was better than other catalysts (Figure 1A). Next, on the basis of TAC-900 as the catalyst carrier, the ratio of glutaraldehyde solution to TA 250:1, and carbonization temperature 900°C, the cobalt loading of the catalyst was optimized (Figure 1B). With the cobalt loading increasing, the catalyst activity increased significantly. Both the conversion of EL and the yield of GVL reached to the highest values when the cobalt loading came to 17 wt%. Further increasing the cobalt loading to 24 wt%, the increasing of the activity was not obvious. Thus 17 wt% cobalt loading was selected for the follow-up researches. The above results showed that the carbonization temperature of the TA precursor had significant effects on the activity of the obtained catalysts. Therefore, the structure of the catalysts was analyzed through a series of characterizations in the following studies.

**TABLE 1:** Catalytic performance of different catalysts in the conversion of EL to GVL.

Entry	Catalyst	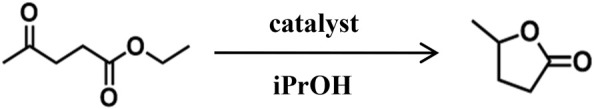					
		Con. (%)	Yield (%)	Sel. (%)	S_BET_ ^a^ (m^2^/g)	V_total_ ^b^ (cm^3^/g)	Pore size^c^ (nm)
1	No catalyst	-	-	-			
2	TAC	-	-	-			
3	CoO	-	-	-			
4	CoCl_2_ 6H_2_O	13.8	0.6	4.4			
5	Co-TAC-300	66.3	53.9	81.2	7.3	0.0123	2.8
6	Co-TAC-500	95.8	76.5	79.9	2.9	0.0116	3.8
7	Co-TAC-600	95.6	79	82.7	6.1	0.0135	3.1
8	Co-TAC-800	95.6	82.7	86.5	5.1	0.0188	2.7
9	Co-TAC-900	97.5	84.1	86.3	6.9	0.0194	2.5
10	Co/TAC-900-air^d^	22.3	2.5	11.1			

a Specific surface area was calculated with the BET, method from the adsorption branch of nitrogen sorption isotherm.

b Volume of pores was estimated from single point adsorption total pore volume of pores.

c Average pore size was calculated with the Barrett‒Joyner‒Halenda (BJH) method from the adsorption branch of nitrogen sorption isotherm.

d Oxidation of the Co/TAC-900 catalyst at 250°C for 3 h under the air atmosphere in the tube furnace.

Reaction conditions: 5 ml iPrOH, 100 mg catalyst, 0.1 MPa N_2_ 150°C, 3 h.

### Catalyst Characterization

**FIGURE 2:**
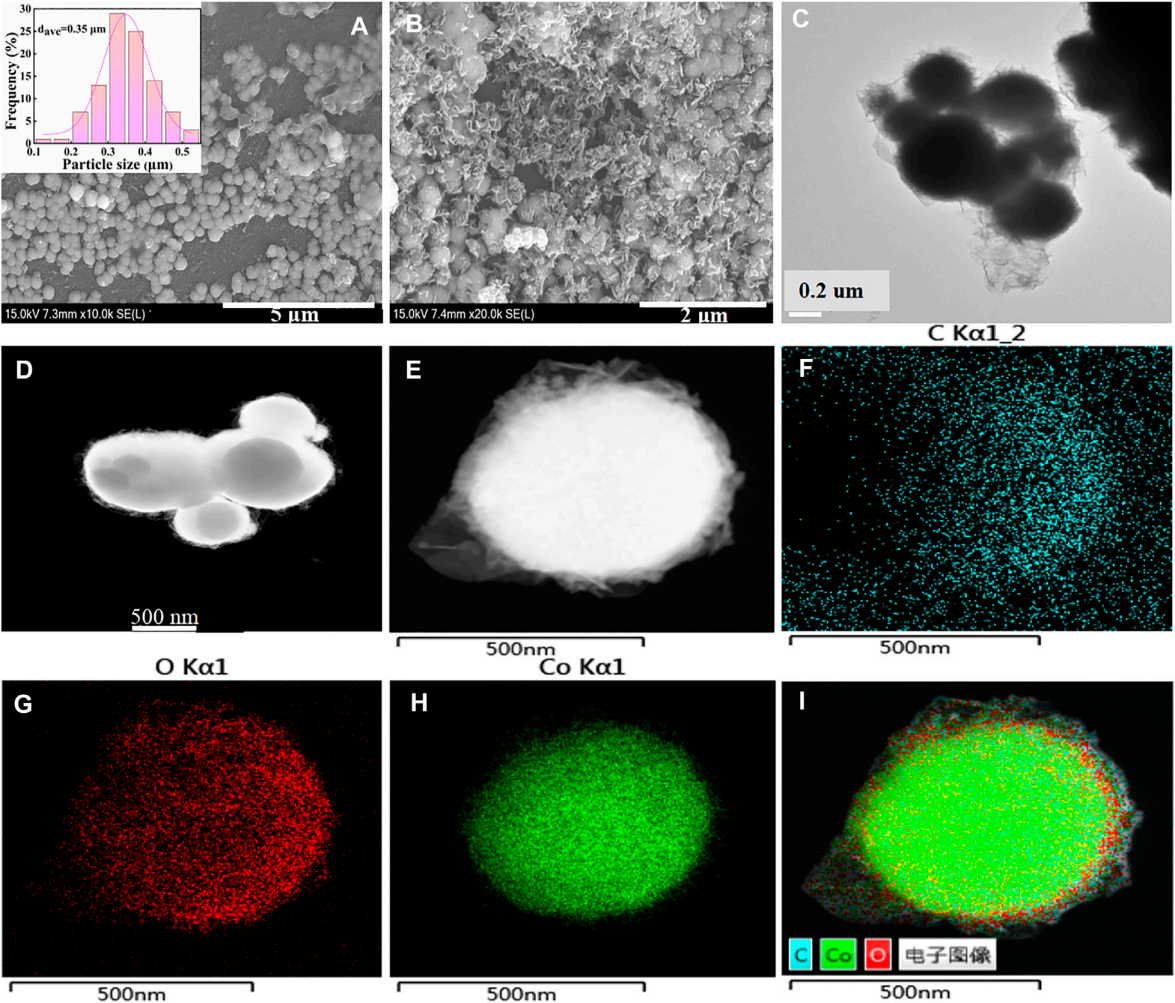
**(A)** and **(B)** SEM images, **(C)** HRTEM image of Co/TAC-900, **(D)** and **(E)** HAADF-STEM images of Co/TAC-900, **(F)** C element, **(G)** O element, **(H)** Co element, **(I)** overlapping map of **(F–H)**.

SEM was used to observe the surface morphology of the catalyst. As depicted in SEM images of Co/TAC-900 (Figure 2A), the catalyst exhibited uniform spheres with an average diameter of 0.35 μm. Characterizing the surface morphology of the spheres under high magnification, it could be seen that certain materials were covered on the spheres (Figure 2B). HRTEM proved that the spheres in the catalyst had a core-shell structure (Figure 2C). In order to give insights into the structures of the catalyst, HAADF-STEM images of Co/TAC-900 (Figures 2D, E) and the corresponding EDX mapping were conducted (Figures 2F–I). HAADF-STEM images showed that the spheres indeed had a core-shell structure, and the EDX mapping showed that cobalt element formed the core, and carbon element formed the shell structure. Besides, O elements existed in the catalyst, and the special distribution of O elements was mainly accompanied by the presence of Co element, indicating that partial cobalt might exist in the form of cobalt oxide, which would be discussed below. The specific surface area and pore size distribution of the Co/TAC-T catalysts were shown in Table 1 (entries 5–9) and Supplementary Figure S1. The specific surface area and pore size of the catalysts at different carbonization temperatures had no significant difference, and there was no obvious change rule. The catalysts at different carbonization temperatures all presented a typical type IV curve with a H_3_ hysteresis loop, indicating that the catalyst was a mesoporous material.

**FIGURE 3:**
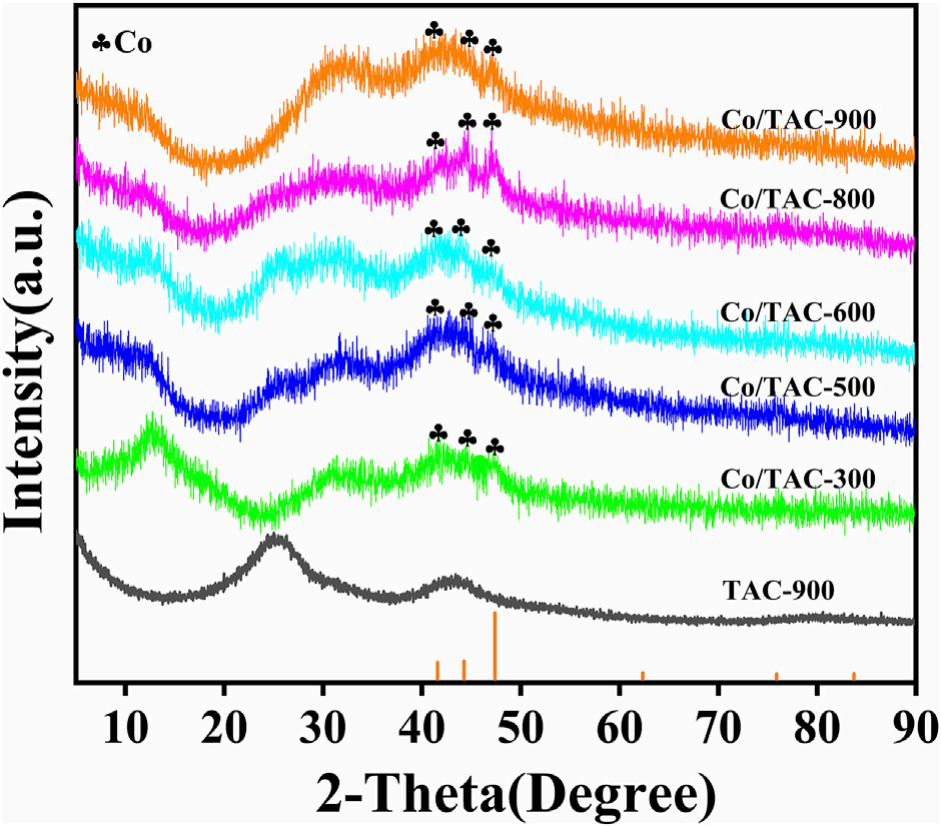
XRD patterns of Co/TAC-T.

The crystal structure of the catalyst was characterized by XRD. As shown in Figure 3. The diffraction peaks at 25.6° and 43.5° of TAC-900 can be assigned to the C (002) and C (001) planes of amorphous carbon (Chen et al., 2021). Compared with the carrier, all the catalysts have obvious diffraction peaks of metallic Co (JCPDS database PDF#89–4308) at around 41.5°, 44.2°, and 47.3°, which were corresponded to the (100) (002), and (101) crystal lines of cubic cobalt, respectively (Xu et al., 2020b; Kong et al., 2021). In addition, no other crystalline forms of cobalt were found in the XRD pattern. This result proved that Co^0^ appeared after the reduction of hydrazine hydrate.

**FIGURE 4:**
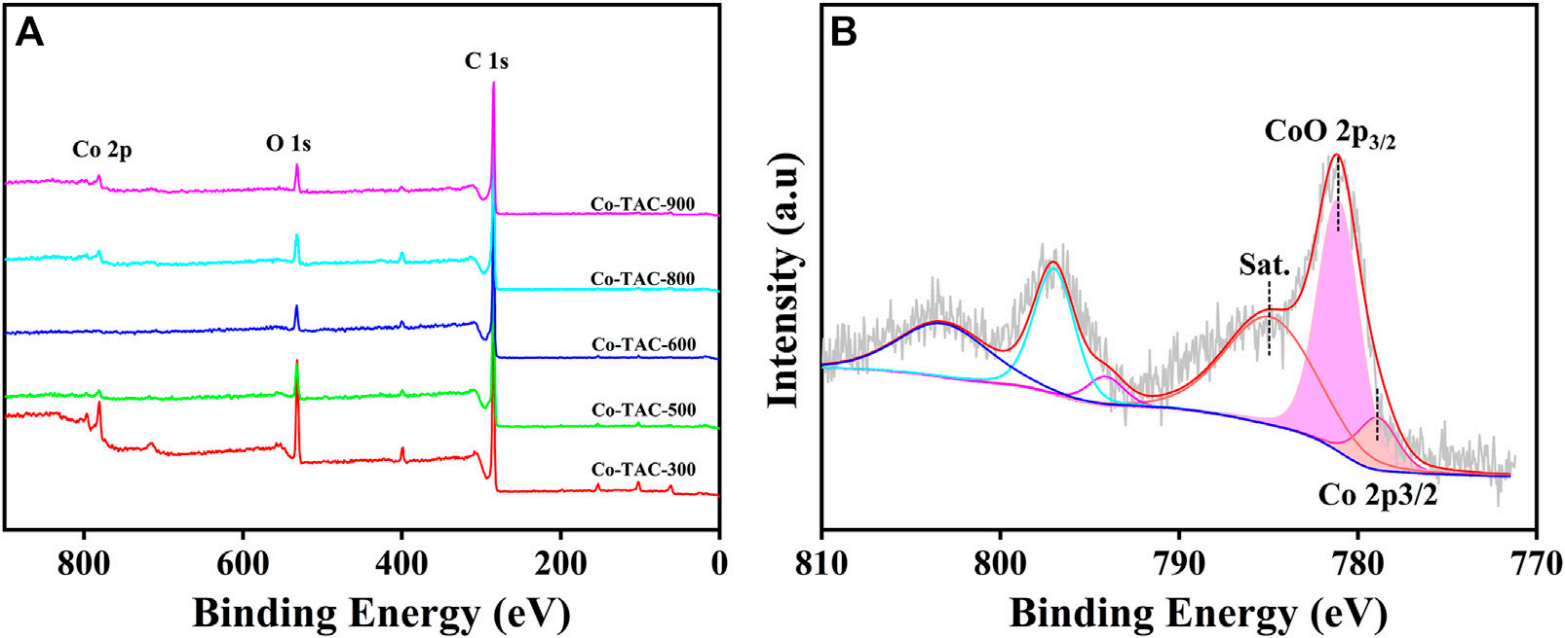
XPS curves of **(A)** survey, **(B)** Co 2p of Co/TAC-900.

In order to analyze the chemical valence of Co species in the catalyst, XPS survey of Co/TAC-T was conducted. The results showed that the presence of Co, C and O elements in the catalyst (Figure 4A). The Co 2p_3/2_ XPS spectrum was fitted into three peaks at 778.8, 780.6, and 784.9 eV, respectively (Figure 4B), corresponding to the metallic Co, CoO and the satellite peak of CoO (Zhao et al., 2019). Cobalt species existed in the Co/TAC-900 catalyst mostly in the form of Co^2+^, and only a small fraction of Co^0^ existed. As shown in Table 2, the relative ratios of cobalt with different valences were analyzed by calculating the ratios of peak areas after peak fitting. The Co 2p peak fitting showed that the metal cobalt content was 16.8%. The ratio of metallic cobalt increased with the increasing of the pyrolysis temperature of the carbon support from 300°C to 900°C, and the higher content of metallic cobalt in the catalyst was beneficial to the activity of the prepared catalysts, which was consistent with the results in Table 1.

**TABLE 2:** Contents of metallic Co and CoO in different Co/TAC-T catalysts.

Entry	Sample	Metallic Co % (778.5 eV)	CoO % (781.1 eV)
1	Co-TAC-300	10.43	89.57
2	Co-TAC-500	11.52	88.48
3	Co-TAC-600	11.61	88.39
4	Co-TAC-800	13.73	86.27
5	Co-TAC-900	16.83	83.17

**FIGURE 5:**
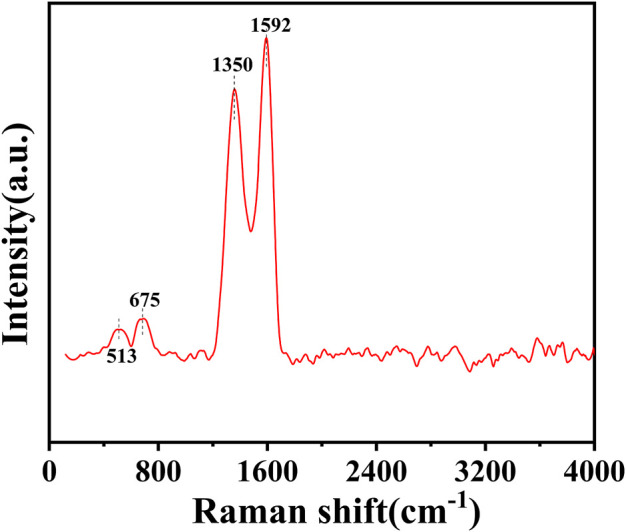
Raman spectra of Co/TAC-900.

The structure of the catalyst was further investigated using Raman spectroscopy. As can be seen in Figure 5, the G band at 1592 cm^−1^ and the D band at 1350 cm^−1^ of the catalyst represent the in-plane vibrations of sp^2^ carbon atoms and the defect-induced imperfect crystal structure, respectively (Chuang et al., 1976). The typical characteristic peaks for CoO appeared at 513 cm^−1^ and 675 cm^−1^ (Song et al., 2018; Liu et al., 2021), which also confirmed the existence of CoO. This result was consistent with the characterization of XPS. Therefore, cobalt species coexisted in the catalyst in the form of Co^0^ and CoO.

The acidity and basicity of the catalyst play a crucial role in the hydrogen transfer reaction (Song et al., 2015a; Li et al., 2018). Therefore, the acidity and basicity of the catalysts were tested using NH_3_-TPD and CO_2_-TPD, respectively. The results were shown in Supplementary Figure S2. Co/TAC catalysts had acid and basic sites, the acidic sites were from Co^2+^, and the basic sites were from O^2-^ (Kong et al., 2021). With the increasing of the carbonization temperature, the desorption peak of the adsorbed gas moved to the high temperature area and the intensity decreased. It could be seen that the total contents of acidic and basic sites of Co/TAC-300, 500 were much stronger than that of Co/TAC-600, 800 and 900. This may be because the increasing of the carbonization temperature led to the change of the support structure and the destroy of the acidic and basic centers in the catalyst, resulting in the decreasing in the content of acidic and basic sites. The acidic and basic sites of Co/TAC-300, 500 were mainly weak to mediumly strong. The contents of the acidic and basic sites of Co/TAC-600, 800, and 900 tended to be stable without significant changes with the increasing of pyrolysis temperature. The quantitative calculation results of the TPD of the catalysts were shown in Supplementary Tables S1, S2. Among them, Co/TAC-900 had more strongly acidic sites but less strongly basic sites compared to Co/TAC-600, 800. The abundant strongly acidic sites may be one of the reasons for the high activity of this catalyst. It has been reported that stronger acid strength but weaker base strength was beneficial to transfer hydrogenation and could suppress side reactions (Song et al., 2015b).

### Optimization of Reaction Conditions

**FIGURE 6:**
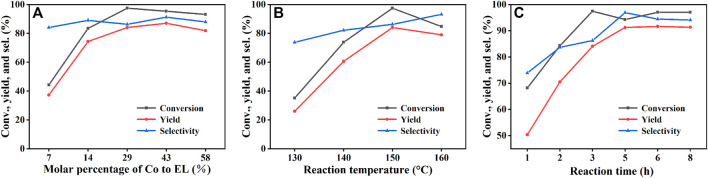
The effects of reaction parameters on the performance of Co/TAC-900: **(A)** molar percentage of Co to EL. **(B)** reaction temperature, and **(C)** reaction time. Reaction condition: **(A)** EL 1 mmol, iPrOH 5 ml, 0.1 MPa N_2_, 150°C, 3 h, **(B)** EL 1 mmol, Co/TAC 100 mg, iPrOH 5 ml, 0.1 MPa N_2_, 3 h, **(C)** EL 1 mmol, Co/TAC 100 mg, iPrOH 5 ml, 0.1 MPa N_2_, 150°C.

In the subsequent studies, the effects of different reaction conditions were investigated to realize the high efficiency of the catalyst. The effects of catalyst dosage, reaction temperature and reaction time on the catalytic performance were studied using Co/TAC-900 as the catalyst prepared under the optimal preparation conditions Figure 6. As shown in (Figure 6A), with the increasing of the catalyst dosage from 25 to 100 mg corresponding to the molar percentage of Co/EL from 7 to 29%, both the conversion of EL and the yield of GVL increased significantly, and nearly total conversion of EL was achieved with GVL yield came to 84.1% under the catalyst dosage of 100 mg. Further increasing the catalyst dosage, the GVL yield had no obvious increasing and even slightly decreased under excessive catalyst dosage. It may be because that too much catalyst increased the viscosity of the reaction solution and hindered the mass transfer during reaction. On the other hand, excessive catalyst dosage also resulted in the waste of the catalyst and the decreasing of utilization efficiency of the catalyst. The reaction temperature was a key factor affecting the catalytic performance of the catalyst (Figure 6B). The conversion of EL and the yield of GVL increased linearly with the increasing of the reaction temperature. When the reaction temperature was 150°C and the reaction time was 3 h, the conversion of EL and the yield of GVL reached to 97.5 and 84.1%, respectively. Further increasing the temperature, the conversion of EL and the yield of GVL decreased slightly, which might be due to the high temperature could affect the microstructure of the catalyst or the adsorption of the substrate on the active sites of the catalyst. Interestingly, the catalyst also showed medium activity at 130°C, indicating that the catalyst could also catalyze the reaction of EL to GVL under milder conditions. It can be seen from (Figure 6C), the conversion of EL and the yield of GVL increased linearly with the extension of reaction time from 1 to 5 h, and the highest conversion and yield could reach to 94.3 and 91.3%, respectively.

### Reusability of the Co/TAC-900

**FIGURE 7:**
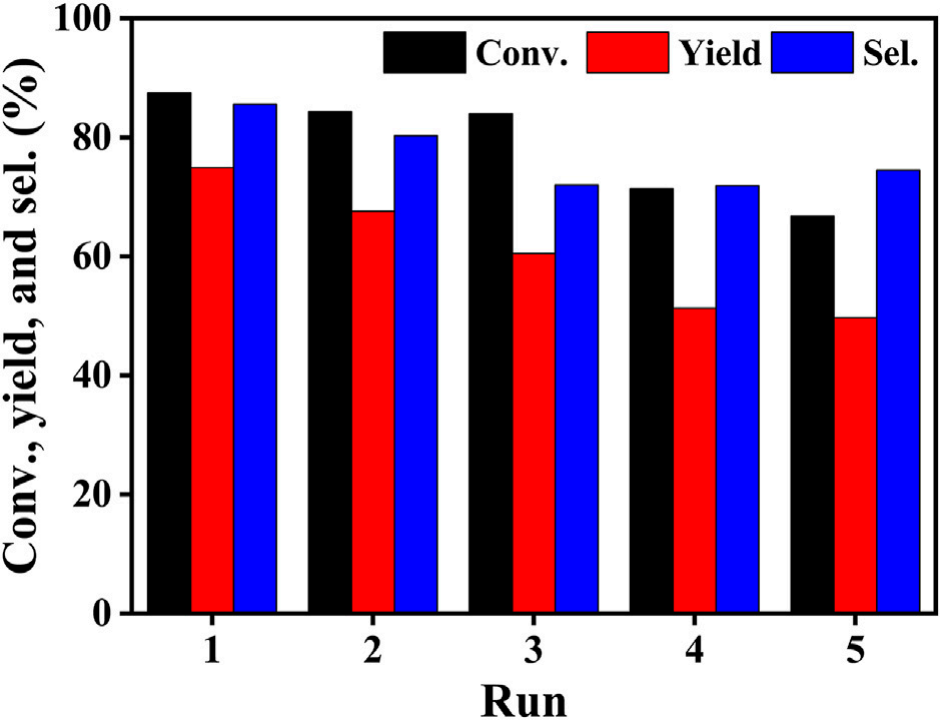
Reusability of the Co/TAC-900 catalyst. Reaction conditions: EL 1 mmol, iPrOH 5 ml, catalyst 100 mg, reaction temperature 150°C, reaction time 2 h.

To examine the stability of the Co/TAC-900 catalyst during recycling, the catalyst was reused for five times. The results showed that conversion, yield and selectivity decreased gradually within five cycles (Figure 7). To further investigate the reasons for the decrease in catalytic activity, the catalysts after five cycles were characterized and compared to the fresh catalyst. The results of ICP (Supplementary Table S3) showed that the cobalt content decreased from 17.0 to 7.7% after five cycles. Next, XPS was used to characterize whether the cobalt element’s valence state of the catalyst changed after recycling, and the results were shown in Supplementary Figure S3. The results showed that the type of cobalt in the catalyst did not change after being recycled for 5 times, but the peak fitting results showed that the content of zero valent cobalt decreased from 16.8% (fresh catalyst) to 5.9% (recycled catalyst). Therefore, we speculated that the reason for the decrease in catalytic activity may be the loss of cobalt and the change of the chemical state of cobalt in the catalyst. This may be related to the structure of the carbon support. It was reported that when the cobalt at the active center was tightly bound to the N atom, the electron distribution on the catalyst surface could be modulated, resulting in excellent stability of the catalyst. N-doping carbon supported cobalt catalysts were reported to be stable (Hasan et al., 2017; Xu et al., 2020b; Chen et al., 2022). It was speculated that the TAC-900 carbon support and the cobalt nanoparticles lacked strong interaction, leading to the easy leaching of cobalt and the changes in chemical state of cobalt. Subsequent studies aiming at increasing the stability of the Co/TAC-900 catalyst were conducted in our group, which will be discussed in another work.

### Substrate Expansion

In order to investigate the catalytic performance of the catalyst for substrates with different structures, the Co/TAC-900 catalyst was used for the hydrogen transfer reaction of different ketone compounds, and the results were shown in Table 3. All ketone substrates could successfully undergo hydrogen transfer under different reaction temperatures, and the conversion of the substrates and the selectivity for the target products were higher than 90 and 95%, respectively. The results proved that Co/TAC-900 had certain universality for the transfer hydrogenation of different ketone substrates.

**TABLE 3:** Co/TAC-900 catalyst catalyzed CTH reaction of ketones.

Entry	Substrate	Product	T (°C)	Time(h)	Conv. (%)	Yield (%)	Sel. (%)
1	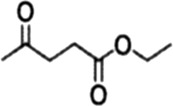	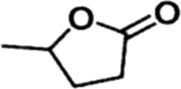	150	5	94.3	91.3	96.9
2	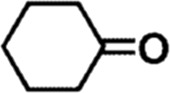	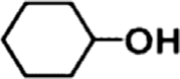	120	15	>99	>99	>99
3	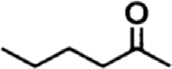	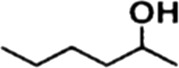	150	8	91.4	86.8	95.0
4	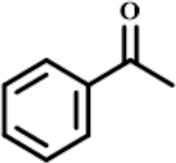	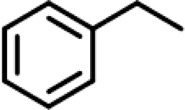	120	12	>99	>99	>99
5	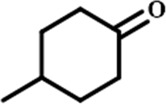	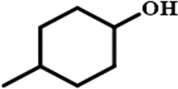	120	12	>99	>99	>99
6	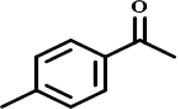	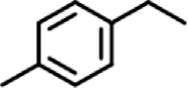	100	12	>99	>99	>99

Reaction condition: substrate 1 mmol, the molar percentage of Co/EL was 29%, iPrOH, 5 ml, 0.1 MPa, N_2_.

The catalytic performance of Co/TAC-900 was compared with other analogue catalysts for hydrogen transfer reactions (Supplementary Table S4). Compared with the catalysts that have been reported in other literatures, the catalysts prepared in this work can obtain relatively high EL conversion and GVL yield under relatively mild conditions (150°C) with medium to higher TOF. In addition, the catalyst was prepared by using tannic acid as the precursor, which was renewable and had potential advantages in catalyst cost.

### Possible Reaction Mechanism

**FIGURE 8:**
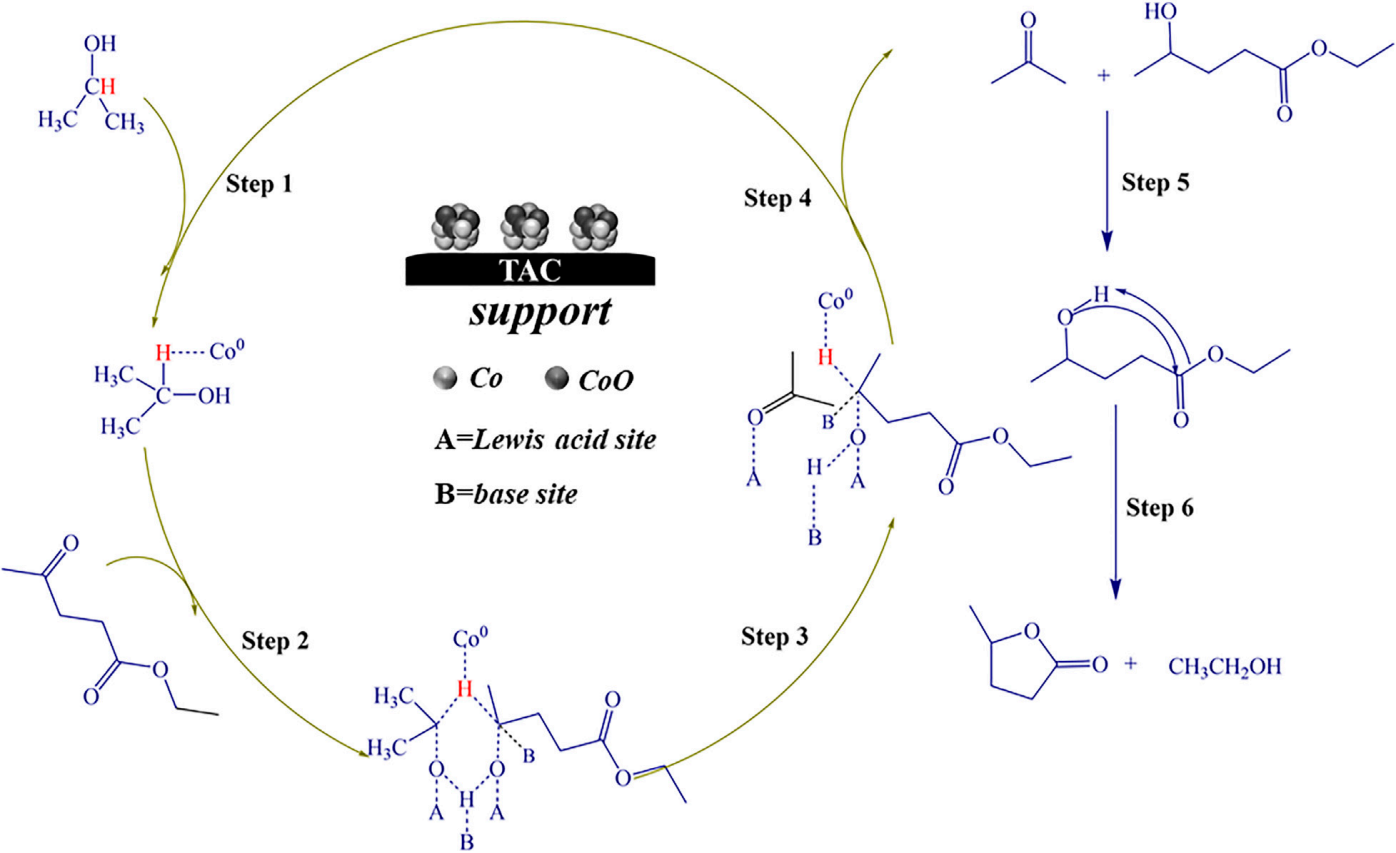
Possible catalytic mechanism of Co/TAC-900 catalyst for the transfer hydrogenation of EL to GVL.

Based on the experimental results and literature reports, the possible catalytic reaction mechanism of Co/TAC-900 catalyst for hydrogen transfer reaction was further discussed. The previous characterization results showed that cobalt species coexisted in the catalyst in the form of Co^0^ and CoO, and the two species cooperated to promote the hydrogen transfer reaction. In the Co/TAC-900 catalyst, CoO provided a Lewis acid-base pair active sites, where the Lewis acid sites were derived from the metal cation Co^2+^, and the basic sites were derived from O^2-^ (Jiang et al., 2020). In the hydrogen transfer reaction using isopropanol as the hydrogen source, the activation of hydrogen in isopropanol was an important step in the reaction process (Geboers et al., 2014). The energy of the 4s orbital in the ground state electron orbital arrangement of Co^0^ was lower than that of the 3d orbital, and electrons preferentially fill the 4 s orbital, while the 3d orbital was not filled. So Co^0^ was more likely to accept electrons and easily adsorbed hydrogen in isopropanol to form Co-H bonds (Song et al., 2015b; Kaźmierczak et al., 2020; Kojima et al., 2021). To prove that Co^0^ plays a leading role in activating H in isopropanol, the Co/TAC-900 catalyst was oxidized in air at 250 °C for 3 h. As a result, as shown in Table 1, entry 10, the catalyst activity after oxidation decreased significantly. To verify the cobalt states of the oxidized catalysts, we carried out XPS characterization for the oxidized catalysts, and the result was shown in Supplementary Figure S4. The result showed that Co^0^ nearly disappeared in the oxidized catalysts. Therefore, it could be concluded that Co^0^ played a critical role in the absorption and activation of isopropanol. Figure 8 showed the possible reaction mechanism of the catalyst for EL conversion. First, Co^0^ activated the hydrogen on the second carbon in isopropanol to form the Co-H bond. Secondly, the hydroxyl groups in isopropanol interacted with the acid-base sites (Co^2+^-O^2-^) on Co/TAC-900, making the hydroxyl groups activated and dissociated to the corresponding alkoxide (Song et al., 2015a; Li et al., 2017b). Meanwhile, the carbonyl group in EL could be adsorbed and activated by Co^2+^-O^2-^ on the catalyst. Then hydrogen transfer took place between the dissociated alcohol and the activated EL *via* a concerted process involving a six-link intermediate to form ethyl 4-hydroxypentanoate (4-HPE). Finally, the 4-HPE is converted into GVL through an intramolecular transesterification under the role of acidic sites in the catalyst. Isopropanol was converted into acetone after losing two hydrogen atoms.

## Conclusion

In conclusion, a novel catalyst was prepared using renewable tannic acids derived carbon as the support and cobalt as the active metal. The effects of the preparation and the reaction conditions on the activity of the catalysts were systematically investigated. The results showed that the prepared catalyst (Co/TAC-900) had a core-shell structure, with cobalt as the core and carbon as the shell. Cobalt existed in the form of Co^0^ and CoO. The pyrolysis temperature had significant influences on the activity of the catalyst *via* changing the acidic and basic properties of the catalyst. The prepared catalyst could fulfill the selective hydrogenation of various ketone compounds with high efficiency, indicating that it had wide substrate universality. The performance of the catalyst decreased during the recycling process, which was proved to be caused by the leaching of the cobalt and the decreasing of the Co^0^ content in the catalyst. Mechanism analysis showed that Co^0^ played a key role during the transfer hydrogenation reaction, and the catalytic reaction was finished by the combined action of Co^0^ together with the acidic and basic sites in the catalyst. This work provides a novel reference for the construction of catalysts in the field of biomass conversion and a potential pathway for the high-value utilization of tannin acid resource.

## Data Availability

The original contributions presented in the study are included in the article/Supplementary Material, further inquiries can be directed to the corresponding author.
