# Supplementary material for: A Novel Tannic Acid-Based Carbon-Supported Cobalt Catalyst for Transfer Hydrogenation of Biomass Derived Ethyl Levulinate
Source: Front Chem. 2022 Jul 11;10:964128. doi: 10.3389/fchem.2022.964128 (PMC9309337; doi:10.3389/fchem.2022.964128)
Supplement: Supplementary file 1 [file DataSheet1.docx]

**Supplementary Material**

**b**

**a**





**Fig.S1.** (a) N_2_ adsorption/desorption isotherms, (b) pore size distribution of Co/TAC-T





**a**

**b**

**Fig.S2.** (a) NH_3_-TPD curves of TAC-900 and Co/TAC-T, (b) CO_2_-TPD curves of TAC-900 and Co/TAC-T.

**Table S1.** NH_3_-TPD quantitative calculation result of Co/TAC-T

| Catalyst | Acidity (mmol g^-1^) | | | | | | | | |
| --- | --- | --- | --- | --- | --- | --- | --- | --- | --- |
|  | T_1_^a^ | Q_1_^b^ | T_2_ | Q_2_ | T_3_ | Q_3_ | T_4_ | Q_4_ | Total^c^ |
| TAC-900 | 154.2 | 0.46 | 552.3 | 0.69 |  |  |  |  | 1.15 |
| Co/TAC-300 |  |  | 408.1 | 24.49 |  |  |  |  | 24.49 |
| Co/TAC-500 |  |  | 585.6 | 12.86 |  |  |  |  | 12.86 |
| Co/TAC-600 |  |  |  |  | 645.5 | 7.10 |  |  | 7.10 |
| Co/TAC-800 | 372.3 | 1.25 | 547.0 | 1.60 | 786.9 | 2.02 |  |  | 4.87 |
| Co/TAC-900 | 356.3 | 1.28 |  |  | 608.7 | 3.47 | 758.8 | 1.85 | 6.60 |

^a^T_1_ represents the temperature points with the strongest absorption value of NH_3_ in the interval 0-400 °C; T_2_ represents the temperature points with the strongest absorption value of NH_3_ in the interval 400-600 °C; T_3_ and T_4_ represent the temperature points with the strongest absorption value of NH_3_ in the interval 600-800 °C, respectively.

^b^ Q_1_, Q_2_, Q_3_ and Q_4_ respectively represent the molar amount of NH_3_ absorbed in each temperature range stated above, respectively.

c “Total” means the total acidic sites, and its value is the sum of Q_1_, Q_2_, Q_3_ and Q_4_.

**Table S2.** CO_2_-TPD quantitative calculation result of Co/TAC-T

| Catalyst | Basicity (mmol g^-1^) | | | | | | | | |
| --- | --- | --- | --- | --- | --- | --- | --- | --- | --- |
|  | T_1_^a^ | Q_1_^b^ | T_2_ | Q_2_ | T_3_ | Q_3_ | T_4_ | Q_4_ | Total^c^ |
| TAC-900 |  |  |  |  | 573.2 | 0.36 |  |  | 0.36 |
| Co/TAC-300 |  |  | 400.3 | 3.27 |  |  |  |  | 3.27 |
| Co/TAC-500 |  |  |  |  | 583.4 | 2.69 |  |  | 2.69 |
| Co/TAC-600 |  |  |  |  |  |  | 624.0 | 1.89 | 1.89 |
| Co/TAC-800 |  |  | 434.2 | 0.47 | 551.5 | 0.25 | 727.3 | 0.77 | 1.49 |
| Co/TAC-900 | 172.2 | 0.13 |  |  | 541.8 | 0.28 | 785.2 | 0.50 | 0.91 |

^a^T_1_ represents the temperature points with the strongest absorption value of CO_2_ in the interval 0-400 °C; T_2_ and T_3_ represent the temperature points with the strongest absorption value of CO_2_ in the interval 400-600 °C; T_4_ represent the temperature points with the strongest absorption value of CO_2_ in the interval 600-800 °C, respectively.

^b^ Q_1_, Q_2_, Q_3_ and Q_4_ respectively represent the molar amount of CO_2_ absorbed in each temperature range stated above, respectively.

c “Total” means the total basic sites, and its value is the sum of Q_1_, Q_2_, Q_3_ and Q_4_.

**Table S3.**Cobalt content before and after cycling tested by ICP-AES

| Sample | Co wt% |
| --- | --- |
| Before recycle | 17 |
| After recycle | 7.7 |





**5.9 %**

**16.8 %**

**Fig.S3.** High resolution XPS spectra of Co 2p about Co/TAC-900 before and after recycle.

**Table S4.** Comparison of different catalysts in literatures.

| Entry | Catalysts | T(℃) | T(h) | Conv.(%) | Yield(%) | Sel.(%) | TOF(h^-1^) | Ref. |
| --- | --- | --- | --- | --- | --- | --- | --- | --- |
| 1 | Co/TAC-900^a^ | 150 | 5 | 94.3 | 91.3 | 96.9 | 0.66 | This work |
| 2 | CoO/C^b^ | 200 | 4 | 91.5 | 90.8 | 99.2 | 0.72 | [1] |
| 3 | Co@OFR^+^C-900 | 150 | 3 | 80.7 | 56.0 | 69.4 | 0.56 | [2] |
| 4 | Co@NGC^b^ | 180 | 6 | 62.0 | 46.2 | 74.5 | 0.34 | [3] |
| 5 | Co(AcO)_2_^b,c^ | 150 | 6 | 12.1 | 8.6 | 71.7 | 0.1 | [4] |
| 6 | Ni-Cu/Al_2_O_3_^d^ | 200 | 4 | 95 | 94.5 | >99 | 0.6 | [5] |

The values of turnover frequency (TOF) were calculated based on the EL mole converted at per mol of active sites and per hour.

^a^ Typical reaction conditions：EL 1mmol,Co/TAC catalyst 0.1 g

^b^ The substrate is furfural

^c^ Hydrogen source is formic acid

^d^ Hydrogen source is 3-pentanol





**Fig.S4**. High resolution XPS spectra of Co 2p about Co/TAC-900 oxidized in air at 250 ºC for 3 h.

**References**

1. Jiang, S., Li, F., Huang, J., Wang, Y., Lu, S., Li, P. et al. (2020). Catalytic transfer hydrogenation of furfural over magnetic carbon‐encapsulated CoO@ C catalyst. *ChemistrySelect* 5, 9883-9892. doi: [10.1002/slct.202002269](https://doi.org/10.1002/slct.202002269)
2. Chen, Y., Yao, X., Zhou, H., He, R., and Liu, Q. (2022). A novel and efficient N-doping carbon supported cobalt catalyst derived from the fermentation broth solid waste for the hydrogenation of ketones via Meerwein–Ponndorf–Verley reaction. *Appl. Catal. A Gen.* 630, 118436. doi: [10.1016/j.apcata.2021.118436](https://doi.org/10.1016/j.apcata.2021.118436)
3. Wu, J., Yan, X., Wang, W., Jin, M., Xie, Y., and Wang, C. (2022). Highly dispersed CoNi alloy embedded in N‐doped graphitic carbon for catalytic transfer hydrogenation of biomass‐derived furfural. *Chem. Asian J.* 16, 3194-3201.doi: [10.1002/asia.202100727](https://doi.org/10.1002/asia.202100727)
4. Xu, L., Nie, R., Lyu, X., Wang, J., and Lu, X. (2020). Selective hydrogenation of furfural to furfuryl alcohol without external hydrogen over N-doped carbon confined Co catalysts. *Fuel Process. Technol.* 197, 106205. doi: [10.1016/j.fuproc.2019.106205](https://doi.org/10.1016/j.fuproc.2019.106205)
5. Kannapu, H. P. R., Mullen, C. A., Elkasabi, Y., and Boateng, A. A. (2015). Catalytic transfer hydrogenation for stabilization of bio-oil oxygenates: Reduction of p-cresol and furfural over bimetallic Ni–Cu catalysts using isopropanol. *Fuel Process. Technol.* 137, 220-228. doi: [10.1016/j.fuproc.2015.04.023](https://doi.org/10.1016/j.fuproc.2015.04.023)
